# Cue-based feeding and short-term health outcomes of premature infants in newborn intensive care units: a non-randomized trial

**DOI:** 10.1186/s12887-021-03077-1

**Published:** 2022-01-06

**Authors:** Sefatbaqa Samane, Zahed Pasha Yadollah, Hasanpour Marzieh, Hajian - Tilaki Karimollah, Zarkesh Mohammad Reza, Arzani Afsaneh, Heidelise Als

**Affiliations:** 1 Master of Newborn Intensive Care Nursing, School of Nursing and Midwifery, Student Research Committee, Babol University of Medical Sciences, Babol, Iran; 2 Non-Communicable Pediatric Diseases Research Center, Health Research Institute, Babol University of Medical Sciences, Babol, Iran; 3 Pediatric and Neonatal Intensive Care Nursing Education Department, School of Nursing and Midwifery, Tehran University of Medical Sciences, Tehran, Iran; 4 Department of Biostatistics and Epidemiology, School of Public Health, Babol University of Medical Sciences, Babol, Iran; 5 Department of Neonatology, Yas Hospital Complex, Tehran University of Medical Sciences, Tehran, Iran; 6 Center for Non-Communicable Pediatric Diseases, Health Research Institute, School of Nursing and Midwifery, Babol University of Medical Sciences, Babol, Iran; 7 Department of Psychiatry, Harvard Medical School, Neurobehavioral Infant and Child Studies, Boston Children’s Hospital, Boston, MA USA

**Keywords:** Infant, Premature, Cues, Nurses, Feeding methods, Outcome assessment, Health care

## Abstract

**Background:**

Feedings based on behavioral cues is a method relying on infants’ behavioral expressions of readiness to feed. The objective of this interventional study was to determine the effect of cue-based feeding on the short-term health outcomes of preterm infants.

**Methods:**

This quasi-experimental study utilized a historical or phase lag design. It involved 60 preterm infants admitted to an Iranian referral hospital’s Level III-Newborn Intensive Care Unit (NICU) from April 2017 until January 2018. The experimental group (*n* = 30) received a three-step intervention of offering behavioral-cue-based oral (BCBO) feedings: Step 1 – One BCBO feeding every 12 hours for 3 days; Step 2 - Two BCBO feedings every 12 h for 3 days; and Step 3 – All feedings as BCBO feedings for 3 days. The control group received standard care feedings. Group difference data were analyzed with SPSS version 16 using descriptive and inferential statistics.

**Results:**

The infants’ mean weight at time of discharge for the intervention and control groups were 1492.79 ± 21.65 g and 1395.71 ± 17.61 g (*P* = .003) respectively. The mean durations of achieving full oral feedings in the intervention and control groups were 17 ± 6 and 20 ± 11 days, respectively (*P* = .19). The mean frequencies of hypoxia were 1 ± 1.54 and 5 ± 9.31 respectively (*P* = .03) and of gavage feedings 725 ± 584 and 1846 ± 2097 respectively (*P* = .009). No apnea events were reported for the intervention group; the frequency of apnea in the control group was 1 ± 2.11 (*P* = .16).

**Conclusion:**

The findings indicate that cue-based feeding is beneficial for preterm infants. Therefore, it is recommended that nurses employ cue-based feeding in the NICU.

**Trial registration:**

IRCTID: IRCT20170828035962N2. Registered 27 may 2018 – Retrospectively registered, https://en.irct.ir/trial/27024.

## Background 

According to the World Health Organization (WHO), infants born before the 37th week of gestational age are considered premature [1]. At present, in the United States, approximately 12% [2] of infants and in Iran 10% [3] of infants are born prematurely. The number of premature infants and their survival rates are increasing worldwide [4]. Preterm infants are at risk for life-threatening illnesses. (e.g.; diseases that affect the gastrointestinal, respiratory, neurological and cardiovascular systems) [5]. In addition, poor muscle tone, immature oromotor control and coordination of sucking, swallowing, and breathing often lead to difficulties in achieving timely independent oral feedings [6]. Browne et al. [7] found that premature infants had problems with successful feeding and weight gain not only during their NICU (Neonatal Intensive Care Unit) stays but also after discharge. Respiratory status, oxygen saturation and heart rate are sensitive indicators of premature infants’ ability to manage feeding successfully [8]. In order to support preterm infants in feeding competence, typically, feeding volumes required for growth are provided through gavage tube feedings, either naso- or oro-gastric, until infants are competent in independent oral feedings [9]. Oral feeding presents a complex accomplishment for most preterm infants [10]. Achieving independent oral feeding is one of the American Academy of Pediatrics’ recommended criteria for infant discharge [11]. Infant feeding protocols, thus, should be evaluated with the goal to assist premature infants in safely acquiring independent oral feeding skills before discharge [12]. Some interventions such as non-nutritive sucking and individualized developmental care have shown to be efficacious in supporting the shift from gavage to independent oral feeding, which in turn likely will reduce the duration of hospital stays [10]. Today, increasing emphasis is placed on the Newborn Individualized Developmental Care and Assessment Program (NIDCAP) in NICUs. This individualized approach to care is based on reading each preterm infant’s behavioral cues, and on formulating a plan of care based there upon. This approach enhances and builds upon the infant’s strengths, and supports the infant in areas of sensitivity and vulnerability [13]. Given the specificity of each preterm infant’s behavioral cues for self-regulation during feeding, [12] a shift from gestational age-based guidelines for initiating oral feedings or volume-based feedings [14] to an approach based on infants’ behavioral cues likely should occur. Behavioral cue-based feeding (BCBF) based on close observation of the infant’s behavioral signals [15] is a method in which caregivers determine how and when an infant expects to be fed. Each infant is considered an individual with meaningful behaviors. A gestational age and volume-driven approach to feeding therefore is shifted towards an infant-driven approach [16]. Crick et al. (2007) found that cue-based feeding was effective in the successful achievement of premature infants’ competence in earlier independent oral feeding [17]. Behavioral cue-based feeding requires coordinated breathing, sucking, and swallowing for behavioral regulation and physiological stability, and as with all behavior, is influenced by the environment and the caregiver [18]. Results of a study in New York that compared infant-driven feeding protocols and routine feedings based on physicians’ prescription orders showed that the infant-driven approach was associated with faster achievement of oral feeding skills, and thereby earlier hospital discharge [19]. NICU nurses are in an excellent position to identify cue-based infant feeding behaviors since they care for infants for extended time periods; this avails them of ample opportunity to observe closely and get to know the infants in their care. With a primary care nursing team, nurses tend to develop close relationships with infants and their families, which in turn enhance their understanding and observation of preferences, strengths, and vulnerabilities of the infant, as well as of subtle and/or unexpected behavioral changes possibly signaling impending setbacks or illness [20]. Despite the growing evidence of the success and importance of this type of feeding, its implementation continues to meet resistance in NICUs, likely because embracing change is often fraught with anxiety of failure; it requires letting go of the security of well-established, routinized infant age and volume-based protocols [17]. Abdul-Aziz et al. [21] in Egypt found that educating mothers in reading their infants’ cues and using these cues in feeding their preterm infants increased gains in weight, and head circumference, and shortened the time to full nipple feeding. Evidence of the effectiveness of cue-based infant-driven feeding in Iran so far is lacking. Many NICUs in Iran begin oral feedings based on postconceptional age and feeding volume, with resultant frequent feeding problems that delay discharge. Therefore, the current study was designed to determine the effects of behavioral cue-based feeding on preterm infants’ short-term health outcomes.

## Methods

### Trial design

Sixty premature infants admitted to the NICU of a large referral hospital in Tehran, Iran, from April 2017 until January 2018 participated in this quasi-experimental study. In order to prevent information spill-over after training in BCBF, a phase lag design was adopted. Phase 1 served as the base line phase (control group) then 2 weeks washout period (nurses training) and after that Phase 2 as the intervention phase (experimental group). The design of our study was non randomized clinical trial and the samples were performed sequentially.

### Participants

The study hospital’s NICU has 23 level III intensive care beds and a staff of 39 full-time nurses. Infant inclusion criteria were as follows: Gestational age, 28 to 36 weeks based on medical records; physiologically stable by onset of study; lack of congenital malformations; discontinuation of all parenteral fluids and intravenous lines; the ability of infants to access their own mother breast milk and cared for in an incubator. Exclusion criteria included: Intraventricular hemorrhage grade III and IV as established by independent ultrasound and/or MRI; necrotizing enterocolitis; Stage II and III bronchopulmonary dysplasia as based on independent X-ray review; sepsis; major medical illness in the mother, e.g. diabetes; lack of weight gain for three consecutive days; current treatment with mechanical ventilation, sedative medication and/or phototherapy. Background variables recorded included demographic and medical sample descriptors as well as various caregiver characteristics as follow: Gestational age at birth, birth weight, gender, Apgar score at one and 5 min, cause of hospitalization, duration of ventilation, and post conceptional age at start of oral feeding. Nurse caregiver characteristics included age, years of experience, full-time or part-time status, and prior successful completion of courses in breastfeeding and/or infant developmental care. Outcome variables collected during all work shifts included: Timing from onset of independent feedings, frequency of independent oral feeding events, frequency of gavage feeding events, and frequency of oxygen desaturation and of apnea events, all to discharge from the hospital. Additionally, in both groups daily weight-gain since study entry until 21 days thereafter was measured because the minimum duration of an infant’s participation in the study was 21 days. Feeding volumes and feeding methods including direct breastfeeding, cup feeding, dropper, syringe, and gavage feedings were recorded for descriptive purposes only. For both groups’ background and outcome variables were collected from each infant’s medical chart after the infant’s discharge from the NICU by the nurse, who cared for, and was familiar with the infant. All participating nurses in both groups were trained in chart review by the researcher.

### Interventions

After completion of the Phase 1(control group) sample intake (based on routine care: Feeding every 3 hours as prescribed by neonatologist), then we have 2 weeks washout period and after that an intervention training phase was instituted. First, the planned intervention of the behavioral cue-based feeding (BCBF) protocol was discussed, and collaboration and cooperation assurances were obtained from all neonatologists and their assistants, and from the NICU nurse manager and the educational supervisor of the nursery. Subsequent training consisted of two workshops for all staff (39 nurses and 5 physicians). The intervention was modelled on the cue-based behavioral oral feeding protocol as proposed by the University of Utah [17]. Workshop content included the definition of cue-based feeding and reasons for its importance; the goals and benefits of this feeding method; training in the recognition of preterm infants’ behavioral cues as spelled out in the NIDCAP methodology [22], and specifically of behavioral cues associated with feeding, such as readiness to feed, signs of hunger, fatigue, and of satiation. Co-author HM. a certified NIDCAP Professional, supervised this training. To remind the nursing staff of the educational workshop contents, posters depicting/describing feeding based behavioral cues were created and made available throughout the intervention period. Infant cues included waking up, moaning, fussing, relaxed facial expression, eyes fully open, becoming upset, crying, bringing hands to the mouth, grasping, rooting, attempts to suck and/or successfully sucking on a pacifier or finger; modulated good tone, limp facial musculature, limp posture of arms, hands and legs of trunk, head droop, and color changes to pale or mottled, among others [17, 22]. After 2 weeks of BCBF training the intervention group infants were admitted to the study and the BCBF intervention began. The attending neonatologist recorded in the chart when the decision was made to discontinue the infants’ intravenous feedings. The intervention subsequently proceeded in three stages: In the first stage one oral feeding, based on the study infant’s behavioral cues, was offered every 12 h; in the second stage two oral feedings, based on the study infant’s behavioral cues, were offered every 12 h; and finally, in the third stage, all oral feedings were based on the study infant’s behavioral cues. Stages one and two each lasted for 3 days, and from the seventh day forward counted from initiation of the intervention, the infant received complete oral feedings. Once the infant, in addition to complete oral feedings, showed stable weight gain for 72 h, he/she was discharged. In the first and second stages, a nurse trained in cue-based feeding fed the infant. In the third stage, the mothers of the Phase 2 study infants also received cue-based education and provided the feeding when they were present in the nursery. Clinical adaptations to the three stages were instituted when an infant failed to show any signs of wakefulness or hunger for up to 4 hours after the previous feeding; in such cases the infant was fed in accordance with the baby’s clinical status (direct breastfeeding, feeding through a cup, a dropper, a syringe, or gavage) to prevent hypoglycemia. Transition from one stage to the next was subject to appropriate weight gain (at a minimum 10 g within 24 h) and the continued absence of apnea, bradycardia and/or oxygen desaturation during feeding. Otherwise, until the infant achieved adequate weight gain, the infant remained at the same stage of feeding as before.

### Measuring instruments

Infant weight in both groups was measured as a routine nursing care in the NICU by trained nurses at the beginning of each morning shift with the infant uncovered and undressed and by using the same digital scale (SEKA, made in China), which has an accuracy of 5 to 10 grams. To determine arterial oxygen saturation and the frequency of a 20 s apnea (accompanied by a bradycardia and or desaturation) [23], Iranian manufactured cardiovascular monitors (SAADAT) were used.

### Outcomes

Data were analyzed using the Statistical Package for the Social Sciences (SPSS), version 16. For the examination of group differences of continuous variables two-tailed T-tests and repeated measures Analysis of Variance (ANOVA) was used in order to check the weight gain to 21 days after meeting criterion for onset of the intervention in both groups. For categorical variables two-tailed Chi-Square tests were used. A two-tailed probability value (P) of < 0.05 was accepted as statistically significant. In Phase 1, the control group infants (30) began their oral feeding every two or 3 hours according to the study nursery’s routine and physician’s order, dependent on post-conceptional age and nursery-protocol-determined volumes/24 h. Attempts at breastfeeding were initiated by 34 weeks post-conceptional age.

### Sample size

The sample size in each group was estimated to be 30 infants in order to achieve an effect size of 0.5, 95% confidence interval with 80% power.

## Results

Somewhat more study infant’s participants in both groups were to be girls (58.35%; *P* < .14). Table 1 shows the results of the background variable comparisons between the control and the intervention group infants. The two groups were comparable in terms of all of the background variables measured (*P* > .05). However, the control group infants were of lower weight at the time they would have met criterion to begin the intervention had they been in the intervention group, than the intervention group (1327 ± 319.38 vs 1262 ± 318.77; *P* < .44).

**Table 1:** Comparison of means and standard deviations of infant demographics in the intervention and control group

Demographic characteristics of infants	Intervention group *N* = 30 Mean ± SD	Control group *N* = 30 Mean ± SD	*P* value
Gestational age (weeks)	30.5 ± 1.96	31.23 ± 2.12	.17^*^
At birth weight(g)	1327 ± 319.38	1262 ± 318.77	.44^*^
One-minute Apgar score	6.73 ± 1.68	7.2 ± 1.82	.30^*^
Five-minute Apgar score	8.96 ± 1.29	9.26 ± 1.14	.34^*^
Duration of ventilator use (days)	11.53 ± 14.49	8.9 ± 14.54	.48^*^
Birth to oral feeding onset (days)	36.5 ± 19.9	28.8 ± 15.8	. 10^*^
Oral feeding onset to discharge (days)	57.2 ± 21.5	48.7 ± 23.9	.15^*^
**Sex**	N (%)	N (%)	
Boy	15 (50%)	10 (33.3%)	.14**
Girl	15 (50%)	20 (66.7%)	

*IndependentT-tests

** Chi-square

Before the educational BCBF intervention, 89.7% of the nurses had not participated in the NIDCAP course; the other 10.3% had passed the course. Following participation in the BCBF workshop, about 59% of the nurses felt encouraged to take the NIDCAP course. In the case of the breastfeeding course, only 28.2% of the nurses had previously passed this course, while after holding the BCBF workshop 46.2% passed this course. Results of the comparison of the short-term health outcomes for the control and intervention groups are presented in Table 2.

**Table 2:** Comparison of means and standard deviations and of frequency counts respectively of the short-term health outcome measures for the intervention and control group

Short-term health outcomes	Intervention group *N* = 30 Mean ± SD	Control group *N* = 30 Mean ± SD	*P* value
Weight gain until 21 days in 2 groups (g)	1492.79 ± 21.65	1395.71 ± 17.61	.003^**^
Frequency of apnea events	.6 ± 1.54	1 ± 2.11	.16^*^
Frequency of oxygen desaturations	1 ± 1.54	5 ± 9.31	.03^*^
Duration of achieving to full oral feeding (days)	17 ± 6	20 ± 11	.19
Frequency of gavage feeding events	725 ± 584	1846 ± 2097	.009

*IndependentT-Test

**ANOVA

**Fig. 1:**
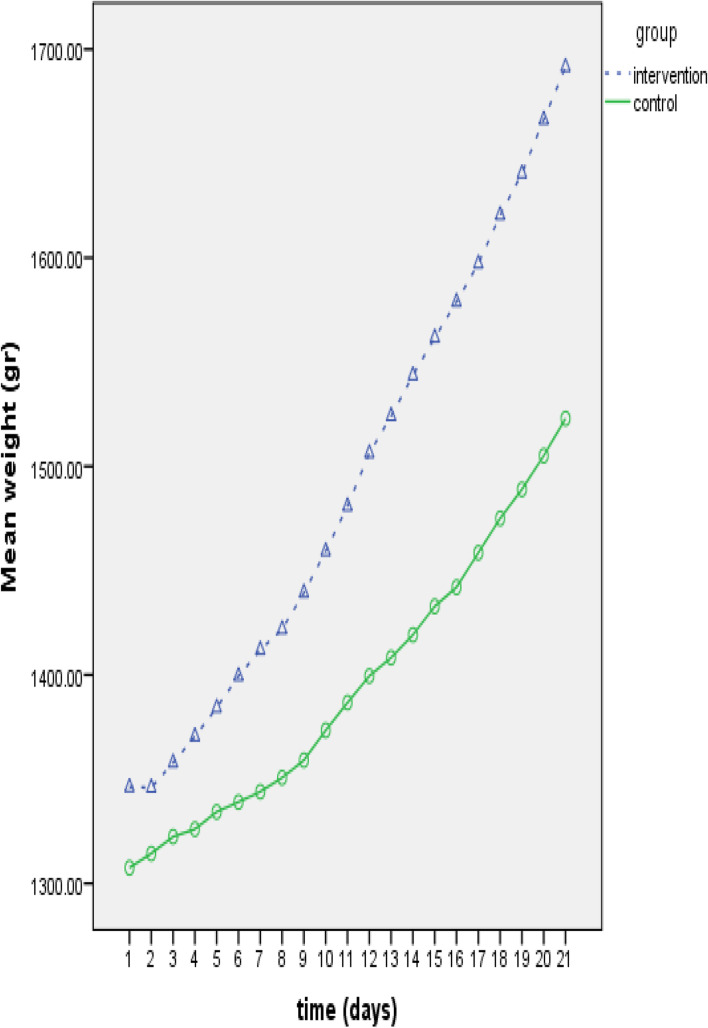
Mean weight gain until 21 days in the intervention and the control group

The frequency of oxygen desaturations (1 ± 1.54 vs 5 ± 9.31; *P* < .035) and gavage feeding events were significantly lower in the intervention group (725 ± 584 vs 1846 ± 2097; *P* < .009) than the control group. On the other hand, the two groups were comparable in terms of the frequency of apneas (.6 ± 1.54 vs 1.0 ± 2.11; *P* < .16) and of the time to achieving full oral feeding (17 ± 6 days vs 20 ± 11 days; *P* < .19), although the results trended in the expected direction. Repeated measure ANOVA showed that mean weight gains from meeting criterion to begin the intervention to 21 days differed significantly between the two groups (*P* = .003). Figure 1 shows that while the intervention and control groups were different at the time of meeting criterion to begin the intervention, the intervention group showed significantly greater weight gain compared to the control group at outcome even after weight at onset of the intervention was entered into the model as a covariate and its effect adjusted with 95% confidence intervals. A more significant difference effect of the intervention was achieved on weight gain over time. This means the interaction effect of time and intervention was significant (*P* < 0.05). In addition, Fig. 1 and our analysis show that a significant trend of weight gain was observed over the time in both groups (*P* < 0.05).

## Discussion

### Interpretation

The results of the study showed that behavioral-cue-based feeding in premature infants demonstrated several positive effects in terms of the infants’ short-term health outcomes. These included greater weight gain, fewer oxygen desaturations and fewer times that gavage feeding was deemed necessary. In line with these results, Abdul-Aziz et al. [21], found that educating mothers in reading their infants’ cues and using these in feeding their preterm infants improved gains in weight and head circumference, and shortened time to full nipple feeding. In the study reported here, the trend of weight gain in the intervention group was higher than that in the control group.

The results of Keshavarz et al. [24], regarding oral stimulation with a pacifier before gavage feeding for support showed that infants’ daily weight gain in the intervention group was significantly better than that of the control group. The intervention in that study was similar to the one studied here; both aimed to improve the achievement of independent oral feeding skills. In contrast, Asadollahpour et al. used Beckman’s oral motor protocol for feeding support of premature infants and found that the two groups were comparable in terms of weight gain. In the reported study reported here, the infants were fed whenever they demonstrated signs of hunger and a tendency to eat. When asleep, the infants were not awakened for routine feedings (e. g. hourly feeding) in order to reduce stressors for the infant [25]. Stress during feeding may increase the number of sensory pathways in the infant’s brain that guide away from feeding and exert adverse effects on the infant’s competence or inclination to feed after NICU discharge [26]. Moreover, the mean frequency of oxygen desaturations in the control group was five times higher than that in the intervention group. These are not only statistically but also clinically meaningful results that add up to improved infant well-being. In congruence with these findings, Pados et al. using a co-regulatory feeding support approach that focused on reduction of environmental stimuli in the NICU, also found that the number of apnea events decreased during feeding, and the approach supported infants to breathe better during feedings. Unlike this current study, however, oxygen desaturation events failed to differ between the control and intervention groups [27]. On average, preterm infants in the behavioral-cue-based feeding group in this study acquired oral feeding skills 3 days earlier than the control group, and although this difference was not statistically significant, it had clinical and economic significance. Earlier discharges can prevent adverse outcomes such as nosocomial infections and reduced medical costs. Other studies also indicated that cue-based fed infants acquired oral feeding skills five to 7 days faster. Newland et al. in the United States performed a study using the cue-based protocol in a level III NICU. The research team consisted of several nurses, physicians and speech therapists. Based on the study outcomes, the NICU changed the former feeding methods such as feeding based on physician’s prescription (volume and time- driven model) [26]. Lessen et al. realized a 5-day reduction in achieving oral feedings by using an oral motor intervention protocol for preterm infants [28]. Also, the studies performed by nurses on infants’ feedings based on behavioral cues showed that oral feedings were achieved earlier in these infants [25]. None of the infants in this study’s intervention group had apnea events once the intervention phase began, whereas in the control group, apnea events occurred in several infants. Although the limitations of this study must be kept in mind, statistically significant and clinically relevant, meaningful advantages became apparent in this pilot study. A randomized controlled trial should be mounted to validate the encouraging findings. Once born out, more extensive nursery wide training and implementation of cue-based care for all care interactions should be considered for the developmental benefits of infants in areas beyond feeding. This likely will support parents, infants, and caregivers alike. It holds promise for positive nursery-wide systems change as supported by the NIDCAP Nursery Program [29]_._

### Limitation

A serious drawback of the current study that requires caution in interpretation of the results, is the quasi-experimental study design employed. A phase lag design is inherently open to criticism since it cannot account for historical changes in the care of infants during the study. While the background variables of the control and intervention groups were comparable, practice change variables during Phase 1 and Phase 2 other than the intervention might account for the results demonstrated. Therefore, it is concluded that as pilot investigation the results of the current study are promising and provide enough evidence to warrant a prospective randomized control trial of the intervention tested. A second drawback of the study was the in-service educator’s variable support to the experimental group infants’ caregivers. With more consistent daily input and support to the intervention group caregivers the results might have been more robust. In a future randomized controlled trial, consistent daily supportive input and guidance to the staff, who cares for the experimental group infants, as well as to the experimental group parents should be considered.

## Conclusion

The results of the study showed that Cue-based feeding (applying behavioral readiness signs and hunger cues to oral feeding management) in premature infants results in greater weight gain, earlier attainment of full oral feeding and fewer oxygen desaturations. Therefore, the implementation of a feeding protocol based on careful observation and understanding of the infants’ behavioral cues should be recommended preliminarily to health care staff and particularly to nurses as well as parents as a guide for all oral feeding. Also further research is needed to examine effects of it on premature infants with specific diseases.

## Data Availability

The data sets used and analyzed during the current study are available from the corresponding author on reasonable request.

## Abbreviations

BCBF: Behavioral-Cue-Based Feeding
NIDCAP: Newborn Individualized Developmental Care and Assessment Program
NICU: Newborn Intensive Care Unit
